# An Improved Technique for Genotyping the *ABCB1* Gene Variant of Exon 21 †

**DOI:** 10.3390/mps6030053

**Published:** 2023-05-26

**Authors:** Johanna Romina Zuccoli, Priscila Ayelén Pagnotta, Viviana Alicia Melito, Jimena Verónica Lavandera, Victoria Estela Parera, Ana María Buzaleh

**Affiliations:** 1 Centro de Investigaciones sobre Porfirinas y Porfirias (CIPYP), CONICET, Hospital de Clínicas José de San Martín, Universidad de Buenos Aires, Buenos Aires 1120, Argentina or melito@qb.fcen.uba.ar (V.A.M.); or vicky@qb.fcen.uba.ar (V.E.P.); or anamaria@qb.fcen.uba.ar (A.M.B.); 2 Departamento de Química Biológica, Facultad de Ciencias Exactas y Naturales, Universidad de Buenos Aires, Buenos Aires 2610, Argentina; priscila.pagnotta@gmail.com; 3 Institute of Biology and Experimental Medicine (IBYME), CONICET, Buenos Aires 2490, Argentina; 4 Cátedra de Bromatología y Nutrición, Facultad de Bioquímica y Ciencias Biológicas, Universidad Nacional del Litoral, Santa Fe 3000, Argentina; chacabuco27@hotmail.com

**Keywords:** *ABCB1* gene, Exon 21, gene variants, genotyping method, single nucleotide variants, restriction fragment length polymorphisms, PCR

## Abstract

The Multidrug Resistance protein (*ABCB1*, *MDR1*) is involved in the transport of xenobiotics and antiretroviral drugs. Some variants of the *ABCB1* gene are of clinical importance; among them, exon 12 (c.1236C>T, rs1128503), 21 (c.2677G>T/A, rs2032582), and 26 (c.3435C>T, rs1045642) have a high incidence in Caucasians. Several protocols have been used for genotyping the exon 21 variants, such as allele-specific PCR-RFLP using adapted primer to generate a digestion site for several enzymes and automatic sequencing to detect the SNVs, TaqMan Allele Discrimination assay and High-Resolution Melter analysis (HRMA). The aim was to describe a new approach to genotype the three variants c.2677G>T/A for the exon 21 doing only one PCR with the corresponding primers and the digestion of the PCR product with two restriction enzymes: *BrsI* to identify A allele and *BseYI* to differentiate between G or T. An improvement of this methodology was also described. The proposal technique here described is demonstrated to be very efficient, easy, fast, reproducible, and cost-effective.

## 1. Introduction

The multidrug resistance gene (*MDR1*, *ABCB1*) encodes for the P-glycoprotein (P-gp), an integral membrane protein of 170 kDA that belongs to the adenosine triphosphate–binding cassette protein superfamily [1,2,3,4,5]. The substrates recognized and transported by P-gp include a variety of drugs. *MDR1* is expressed in many normal tissues. P-gp acts as a cellular efflux pump to control the intracellular concentration of substances being an effective cellular protector against toxic substances. P-gp and other ABC transporters play a key role in absorption, distribution, metabolism, and elimination of drugs, working as a barrier to the entry of compounds into the body or controlling their transfer rate between tissues and compartments [6,7,8,9,10,11,12,13,14]. The discovery of genetic variations that influence the function or expression of P-gp may have a direct impact on the therapy of several diseases and in a near future in the personalized therapy.

There has been described more than 50 SNVs, three of high frequency in exons 12, 21 and 26 that affect the activity and/or expression of de P-gp protein and the toxicity of the drugs [3,4,9,11,15].

The c.2677G>T/A (rs2032582) SNV in exon 21 is a change in the 2677 position of a G for a T or A. Its sequence is CTAGAAGGT [G/T/A]GGGAAGGT. The wild type G variant encodes for Alanine at position 893, while T variant does so for Serine and A for Threonine. T and A variants alter the transporter function respect to G (wild type) variant, affecting drugs pharmacokinetics [16]. Several studies have linked this variant to a different response of pharmacological treatments for diseases such as epilepsy, HIV, coagulation disorders and different types of cancer [17,18,19,20]. Moreover, an exhaustive investigation about rs2032582 genotypes and haplotypes of the *ABCB1* gene was reported [21,22,23,24].

Pagnotta et al. [25] showed the relevance of the T allele in c.2677G>T/A variant in the triggering of Porphyria Cutanea Tarda (PCT) in patients with HIV, possibly through a mechanism involving antiretroviral therapy that are substrates of P-gp. The study was also performed in patients with Acute Intermittent Porphyria (AIP), with a major frequency of A allele in AIP individuals [26]. A bioinformatic analysis was performed using different databases to establish the role of this variant in the PCT-HIV association and the relationship with drugs used in HIV therapy, or those avoided for patients with AIP [27]. 

In an Egyptian population of patients with chronic myeloid leukemia, the frequency of the rs2032582 TT genotype was fewer than that in healthy individuals; moreover, the authors reported that in individuals under therapy with imatinib, the TT genotype was associated with a complete hematological response and also, it has been assigned a role of producing protective factors against drug resistance [28].

Several protocols have been described to genotype c.2677G>T/A variant in exon 21, like allele specific PCR [29], RFLP using adapted primer to generate a digestion site for several enzymes [30] and automatic sequencing [31]. Another genotyping approach used for this variant is the TaqMan Allele Discrimination assay that involves real-time PCR and specific probes [32,33]. Moreover, High-Resolution Melter analysis (HRMA) is a method based in high-resolution melting, a novel, closed-tube post-PCR method to analyze genetic variations in PCR amplicons prior to or as an alternative to sequencing [34].

In our method, genotyping was performed using PCR-RFLP for amplifying the c.2677G>T/A variant in exon 21 with specific designed primers followed by the digestion with two restriction enzymes to characterize the SNVs in exon 21 from genomic DNA.

The technique proposed would be useful for researchers studying *ABCB1* gene variants to underlying the mechanisms that lead to the effectiveness of a xenobiotic used as therapeutically drug for diseases.

This study was conducted to design a new approach that allows us to genotype the three variants of allele 21 by performing only one PCR with specific primers and the use of two restriction enzymes. This proposal technique was very efficient, easy, fast, reproducible, and cost-effective. Moreover, an improvement of this methodology was also suggested.

## 2. Experimental Design

### 2.1. Equipment

- Thermal Cycler
- Thermostatic digital dry bath
- Electrophoresis Tank and Power supply

### 2.2. Biological Material

The recruited cohorts consisted of Caucasian individuals of both sexes. Samples were collected from patients attending the Research Center on Porphyrins and Porphyrias (CIPYP) (Buenos Aires, Argentina) between March 2010 and December 2018. All individuals provided signed consent for participation. The present study conformed with the guidelines stated in the Declaration of Helsinki (www.wma.net/policies-post/wma-declaration-of-helsinki-ethical-principles-for-medical-research-involving-human-subjects, 18th WMA General Assembly, Helsinki, Finland, June 1964 and amended by 64th WMA General Assembly, Fortaleza, Brazil, October 2013) and was approved (June 2013 and certified again in July 2015) by the Institutional Research Ethics Committee of the CIPYP, National Scientific and Technical Research Council.

DNA samples obtained from whole peripheral blood with EDTA were extracted, using the Illustra blood genomicPrep Mini Spin kit (Invitrogen; Thermo Fisher Scientific, Inc.; Waltham, Massachusetts, USA). The amount of genomic DNA obtained was fluorometrically quantified using Qubit Fluorometric Quantification System.

## 3. Procedure

**Validation:** This method was initially carried out by triplicate in 10 DNA samples and after that, it was performed in more than 450 DNA samples [26,27,35,36,37,38].

### 3.1. Primers Design

Use the DNASTAR software (LaserGene) to design the forward and reverse primers. This program led to determine in silico the primer length, length difference of primer pairs, PCR product length, GC proportion, melting temperature (Tm), GC clamp, dimer (including cross-dimers and self-dimers), hairpin structure, and specificity. All these properties are showed in Table 1. Moreover, a target SNV can be discriminated by digestion using available restriction enzymes. The *BrsI* restriction enzyme could differentiate the presence of the A allele from the G allele. If the A allele is present, the digestion product consists of three bands of 491 bp, 433 bp and 177 bp; while allele G/T shows two bands of 668 bp and 433 bp. *BseYI* restriction enzyme produced only two bands of 615 y 486 bp when the G allele is present or one band of 1101 bp for the T allele.

### 3.2. Genotypic Evaluation

**Figure 1:**
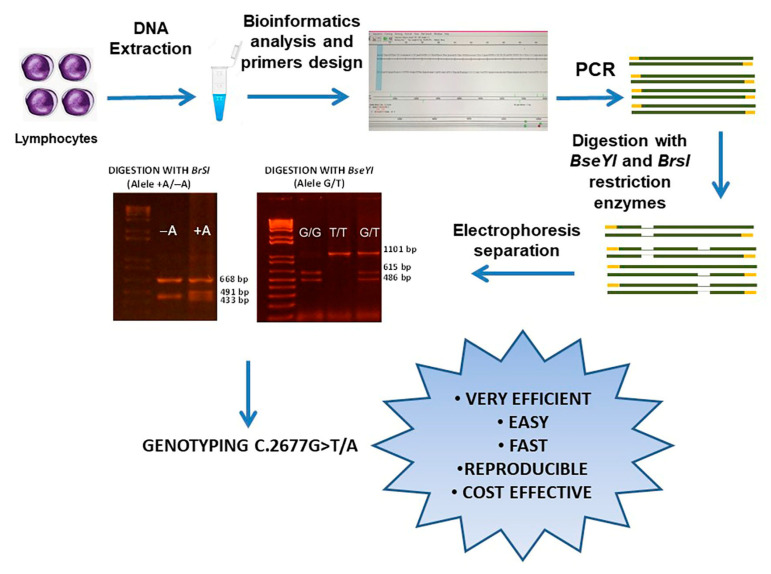
Work scheme of the proposed method.

Genotyping is performed using PCR-RFLP (Figure 1), amplifying a PCR product that contains the c.2677G>T/A variant in exon 21 with specific designed primers using the SeqBuilder and PrimerSelect (LaserGene) programs: Primer Forward: 5′GCTTTAGTAATGTTGCCGTGAT3′, Primer Reverse: 5′ATACCCCTAGCATTTTTCCATA3′. Prepare the PCR reaction mixture in a total volume of 25 µL, containing 14 mM Tris-HCl; 2 mM MgCl_2_; 0.2 mM dNTPs; 0.52 μM of each primer, 2.5 units of Taq DNA polymerase and 10 μL of template DNA (100–500 ng). The genomic DNA sample must have good integrity, quality, and concentration. Make sure to quantify DNA concentration of the samples and perform a horizontal agarose gel electrophoresis to check the quality and integrity of the genomic DNA. Carry out PCR in a Thermal Cycler with an initial denaturation for 3 min at 95 °C followed by 35 cycles of denaturation at 94 °C for 1 min, annealing at 58 °C for 30 s, and extension at 72 °C for 2 min. Perform terminal elongation at 72 °C for 10 min. The total time needed in the performance of the PCR is 2:26 h. Evaluate the 1101 bp PCR product on 1.5% agarose gels using ethidium bromide (30 min, 100 V) as the stain and under the Gel Imaging system.

To identify the A allele, digest the PCR product with the *BrsI* restriction enzyme (New England Biolab, Ipswich, Massachusetts, USA) during 2 h at 65 °C. Analyze the resulted product in 2.5% agarose gels dotted with ethidium bromide (40 min at 80 V). The digestion pattern has three bands of 491 bp, 433 bp y 177 bp for the wild type (G/G). The pattern for G/T is about two bands of 668 bp and 433 bp. To identify the G/T alleles, digest the PCR product with *BseYI* restriction enzyme (Invitrogen) during 1 h at 37 °C, in parallel. Then, analyze the results on an agarose 2.5% gel electrophoresis stained with ethidium bromide (40 min, 80 V). The allele G pattern shows two bands of 615 bp and 486 bp and the T allele pattern shows only 1 band of 1101 bp.

To validate this method, the results were confirmed using Sanger’s automatic sequencing of the PCR product (Sequencer AB13730XL, Macrogen, Seul, South Korea), also known as Dideoxy chain termination sequencing. This methodology involves the incorporation of chain-terminating nucleotides into a complementary DNA strand to a single-stranded DNA template. The chain-terminating nucleotides are radioactive or fluorescent labelled; the detector of the equipment can discriminate the label to determine the nucleotide (A, T, G, C) in the DNA sequence. 

### 3.3. Data Analysis

The bands obtained in agarose gels were classified as GG, GT and TT, +A or -A depending on the restriction enzyme used. The spherograms obtained via Sanger sequencing were manually analyzed. In order to setup the results of our technique, RFLP results were contrasted to that obtained with Sanger sequencing. 

## 4. Expected Results

**Figure 2:**
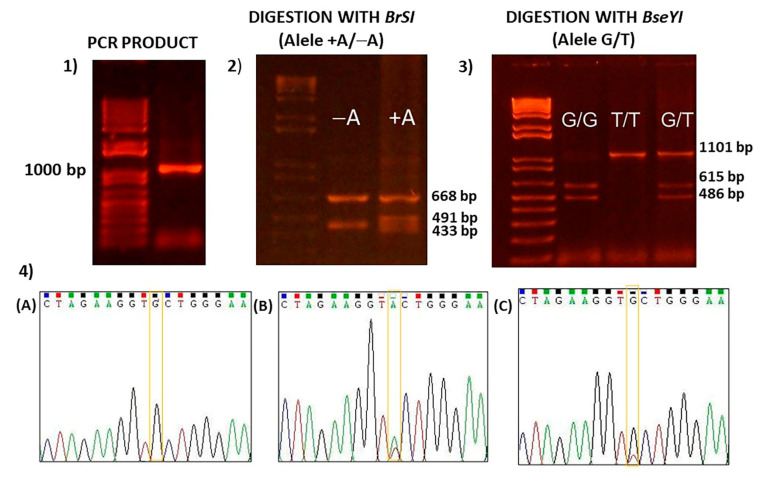
Band pattern of c.2677G>T/A in exon 21 of *ABCB1* gene after amplification and digestion with restriction enzymes. (**1**) PCR product. Line 1: Marker, line 2: Band corresponding to PCR product (1101 bp). (**2**) Digestion with *BrSI* restriction enzyme of PCR product: Line 1: Marker; line 2: Pattern band showing the absence of A allele; line 3: Pattern band for A allele in heterozygosis. (**3**) Digestion with *BreYI* restriction enzyme of PCR product: Line 1: Marker; line 2: Pattern band showing G/G allele in homozygosis; line 3: Pattern band showing T/T allele in homozygosis; line 4: Pattern band showing heterozygosis genotype. (**4**) Sanger’s automatic sequencing of the PCR product (**A**) and after digestion with *BrSI* (**B**) or *BreYI* (**C**) restriction enzymes.

Figure 2 shows the band pattern of c.2677G>T/A in exon 21 of the *ABCB1* gene after amplification and digestion with the two restriction enzymes.

### 4.1. Improvement of the PCR Technique

If DNA samples are of low quality, low concentration or even degraded DNA is present, you can solve these problems using MyTaq™ Extract-PCR Kit (Bioline, London, UK). This kit offers a rapid, easy, and safer alternative for the extraction and amplification of DNA with high specificity because this method incorporates a polymerase that exhibits increased affinity for DNA, thereby improving yield and that uses antibody hot-start technology to avoid non-specific amplification. Moreover, we facilitate PCR set-up because the kit includes all the reagents needed for PCR and a red dye that allows a direct gel loading when electrophoresis is performed.

### 4.2. Discussion

The developed method allowed us to genotype the three variants of exon 21 doing only one PCR with the corresponding primers and then digest the PCR product with two restriction enzymes. This technique demonstrated to be very efficient, easy, fast, reproducible, and cost-effective (Figure 2).

The main differences with other published methods are described below.

Cascorbi et al. [30] described an indirect method of genotyping for which it is necessary to use mismatch primers for the PCR generating two restriction sites for *TaqII* or *BanI* enzymes in the presence of the mutation.

Zoto et al. [29] used two different pair of primers with which two PCR products were obtained to identify the variants G or T.

Zhang et al. [31] used a specific allelic PCR to identify the three variants (G, T or A). 

Other authors employed the TaqMan^®^ PCR technique that consist of pre-optimized PCR primer pairs and two probes to amplify and detect specific alleles in genomic DNA (gDNA) [32,33]. This technique requires to perform a real-time PCR and the synthesis of specific probes, as well as a high-throughput real-time PCR instrument and also trained technical staff, which makes the determination more expensive and not accessible for all the laboratories.

Another technique used in the allelic discrimination of the rs2032582 variant is the HRMA assay, a post-PCR analysis method based on detecting small differences in PCR melting curves, which is specific and sensitive enough to distinguish nucleic acid variations [34]. Although this method is faster, simple, and less expensive than alternative approaches requiring labeled probes, it is necessary to have a high-resolution melting instrument and a master mix of reagents that increases the cost of each determination. Moreover, the performance and data analysis of HRMA results can be affected by type and quality of DNA source material, different isolation methods (DNA preparation), poor melting curve resolution and PCR optimization (primer design) and other issues [39].

The frequencies obtained using the described technique in Argentinean population (T allele: 0.52; A allele 0.03) [25] were similar to those reported for Caucasians (T allele: 0.39–0.43; A allele: 0.02–0.05) [1,29,30,40].

In conclusion, the main difference with the mentioned authors was that they used more complex and expensive methods, which need more time to achieve the result than our proposed technique.

## 5. Reagents Setup

Solutions: All reagents were of molecular grade.

Kit GFx Genomic blood DNA purification (Invitrogen: Waltham, Massachusetts, USA).

10× Tris-HCl buffer.

50 µM oligonucleotide primer forward: 50 pmol/µL in sterile H_2_O (store at −20 °C).

50 µM oligonucleotide primer reverse: 50 pmol/µL in sterile H_2_O (store at −20 °C).

Template DNA: 10 µL human genomic DNA.

25 mM 4 dNTP mix.

2 mM MgCl_2_.

2.5 U/µL Taq DNA polymerase (native or recombinant).

*BrsI* restriction enzyme (New England Biolab, Ipswich, MA, USA)

*BseYI* restriction enzyme (Invitrogen, Waltham, MA, USA)

Agarose 2.5% gel electrophoresis stained with ethidium bromide.

MyTaq™ Extract-PCR Kit (Bioline, London, UK) (optional).

## Figures and Tables

**Table 1:** Main characteristics of the designed primers.

**Forward Primer:** MDR9_New **Reverse Primer:** MDR10_New				5’ATACCCCTAGCATTTTTCCATA3’ 5’GCTTTAGTAATGTTGCCGTGAT3’		
DNA 250 pM, Salt 50 mM				**Forward Primer**	**Reverse Primer**	
Primer Tm Primer Overall Stability Primer Location				50.7 °C −40.0 kc/m 23.44	49.5 °C −40.3 kc/m 1123.1102	
Product Tm – Primer Tm Primers Tm Difference Optimal Annealing Temperature				23.6 °C 1.2 °C 51.1 °C		
Product Length Product Tm (%GC Method) Product GC Content Product Tm at 6xSSC				1101 bp 73.1 °C 33.6% 94.7 °C		
**Product Melting Temperature (%GC Method)**						
**Salt**			**Formamide**			
mM	xSSC	xSSPE	0%	10%	20%	50%
1 10 50 165 330 500 1000	0.005 0.051 0.256 0.846 1.692 2.564 5.128	0.006 0.062 0.312 1.031 2.062 3.125 6.250	44.9 61.5 73.1 81.7 86.7 89.7 94.7	38.4 55.0 66.6 75.2 80.2 83.2 88.2	31.9 48.5 60.1 68.7 73.7 76.7 81.7	12.4 29.0 40.6 49.2 54.2 57.2 62.2

Tm: Melting Temperature. SSC: Saline Sodium Citrate Buffer. SSPE: Saline Sodium Phosphate EDTA Buffer.

## Data Availability

All data generated or analyzed during this study are included in this published article.
